# Trends in the Utilization of Sodium Hyaluronate Eye Drops, Including Disposable and Multiuse Forms, in South Korea: A 14-Year Longitudinal Retrospective Cohort Study

**DOI:** 10.3389/fphar.2020.00720

**Published:** 2020-05-15

**Authors:** Kyung-Bok Son

**Affiliations:** College of Pharmacy, Ewha Womans University, Seoul, South Korea

**Keywords:** eye drop, trends in eye drop utilization, health financing, equity in utilization, cohort study, South Korea

## Abstract

**Introduction:**

Sodium hyaluronate eye drops are frequently prescribed for dry eye disease in South Korea.

**Objectives:**

This study analyzed the trends in the utilization of sodium hyaluronate eye drops and evaluate the impact of the introduction of high-priced disposable forms in the South Korean market.

**Methods:**

The yearly claims data for sodium hyaluronate eye drops from 2002 to 2015 were retrieved from the National Health Insurance Service-National Sample Cohort. Prescriptions of sodium hyaluronate eye drops were sorted by the characteristics of patients and health care institutions.

**Results:**

The number of prescriptions has continuously increased and the share of disposable forms in total prescriptions reached 37% in 2015. Particularly, the prevalence of prescriptions (general users) has increased during the study period from 2,562/100,000 persons in 2002 to 14,732/100,000 persons in 2015, while the incidence of prescriptions (new users) has remained steady during the study period, approximately 3,500/100,000 persons. More female patients were noted in terms of general users and new users, and the proportion of male patients was higher in new users than in general users. The average age of general users increased during the study period, while that of new users slightly decreased. Finally, the distribution of prescription category was significantly different between sex and age groups in frequently prescribed users.

**Conclusions:**

Eye drops in disposable forms, which are safe and more convenient to use, have expanded the market in South Korea and caused equity issues in utilization. Thus, the utilization of eye drops should be closely monitored from the perspectives health equity.

## Introduction

Dry eye disease (DED) is the most common ocular disease causing patients to visit ophthalmology clinics (Lemp, 1995; Lemp and Foulks, 2007; Vehof et al., 2014; Craig et al., 2017). DED is a multifactorial disease of the ocular surface and tears that is accompanied by the inflammation of the ocular surface and increased osmolality of the tear film (Baudouin, 2001; Lemp and Foulks, 2007; Lemp et al., 2007; Vehof et al., 2014; Bron et al., 2014; Craig et al., 2017). The symptoms of DED vary from mild discomfort to severe pain that might disrupt activities of daily living and negatively affect quality of life. Thus, DED has been recognized as an important health issue from the perspectives of ophthalmology and public health (Gayton, 2009; Alves et al., 2013; Bron et al., 2014; Sharma and Mather, 2014; Messmer, 2015; Findlay and Reid, 2018). For instance, a number of studies have reported the prevalence of DED to range from 4.3 to 73.5% (Lekhanont et al., 2006; Uchino et al., 2008; Jie et al., 2009; Han et al., 2011). The variance in the prevalence of DED is caused by the lack of a standardized diagnostic definition and differences in the study population. However, risk factors for DED, such as old age, female sex, and physical environment, including air pollution and indoor air, have been consistently reported in previous literature (Bron et al., 2007; Gayton, 2009; Craig et al., 2017; Findlay and Reid, 2018).

The management of DED is complicated due to the multifactorial characteristics of DED, and the aim of DED management is to restore the homeostasis of the ocular surface and tear film (Hyon et al., 2014; Foulks et al., 2015; Craig et al., 2017; Findlay and Reid, 2018). Thus, a number of therapies might be recommended to identify and treat the primary source of DED. Particularly, the Tear Film & Ocular Surface Society (TFOS) recommended the staged management and treatment of DED, which begins with low-risk and easily accessible therapies, including over-the-counter or prescribed lubricants, and progresses to more advanced therapies for severe symptoms (Craig et al., 2017). Similarly, Korean guidelines for the diagnosis and management of dry eye recommend various therapies according to the severity of DED (Hyon et al., 2014). Particularly, patient education, environmental control, and artificial tears are recommended for patients with mild to moderate levels of DED (Hyon et al., 2014; Craig et al., 2017).

In South Korea, sodium hyaluronate eye drops are prescribed for patients with corneal epithelial disorders caused by endogenous and exogenous diseases and are reimbursed under the National Health Insurance Services (NHIS). Endogenous diseases include Sjögren's syndrome, cutaneous mucosal syndrome (Steven-Jones syndrome), and dry eye syndrome, while exogenous diseases are caused by surgery, chemicals (including drugs), or the wearing of contact lenses. The majority of these patients are prescribed eye drops for dry eye syndrome (Kim et al., 2007; Kim, 2018). Currently, two types of sodium hyaluronate eye drops are available on the market: disposable and multiuse forms. The two types of eye drops could be distinguished by the presence of ophthalmic preservatives, which are frequently used for antibacterial effects (Foulks et al., 2015; Jones et al., 2017).

Ophthalmic preservatives have been recognized as a risk factor for corneal and conjunctival inflammation, indicating that preservative-free eye drops reduce iatrogenic dry eye (Pisella et al., 2002; Jaenen et al., 2007; Leung et al., 2008; Foulks et al., 2015; Craig et al., 2017). Thus, combined formulations to reduce the total amount of preservative are recommended for glaucoma patients who require multiple eye drops. A disposable form of preservative-free eye drops has been available on the market since 2003 to address this issue in South Korea. However, the price of disposable forms is more expensive than that of multiuse forms, implying that frequent use of disposable forms might cause burden for the insurer and patients.

This study has two aims. First, we will analyze the trends in the utilization of sodium hyaluronate eye drops in South Korea from a large-scale population-based dataset. With this analysis, we could approximate the prevalence of DED in South Korea. Second, we will evaluate the impact of the introduction of high-priced disposable forms in the South Korean market from the perspective of health financing and equity in utilization. To this end, we will analyze prescriptions including sodium hyaluronate eye drops, patients who were prescribed medicines, and health-care institutions prescribing medicines to understand for whom, under what conditions, and how eye drops in disposable forms are prescribed.

## Materials and Methods

### Data Source

The National Health Insurance Service-National Sample Cohort (NHIS-NSC), a population-based cohort established by the NHIS, was used for this study (Lee et al., 2017). The NHIS-NSC is composed of 1 million individuals, approximately 2% of the total population of South Korea. The NHIS established a target population of 46,605,433 individuals in 2002, and then 1,025,340 participants were randomly selected from the target population (Lee et al., 2017). Particularly, the NHIS conducted systematic stratified random sampling with proportional allocation within each stratum to construct the NHIS-NSC. Detailed methodologies of the sampling method have been reported previously (Lee et al., 2017).

The NHIS-NSC consists of four databases, including information on participants and their medical treatments and health examination, and information on health-care institutions, for the period from January 2002 to December 2015. The database on participants includes demographic and socioeconomic characteristics of participants, such as the age, sex, level of income, and types of health insurance. The database on medical treatments covers prescriptions, such as the date and duration of the prescription, the prescribed drug's international nonproprietary name, the dosage, and the prescribing health-care institution. The database on health-care institutions includes types and locations of health-care institutions.

### Study Design

**Figure 1:**
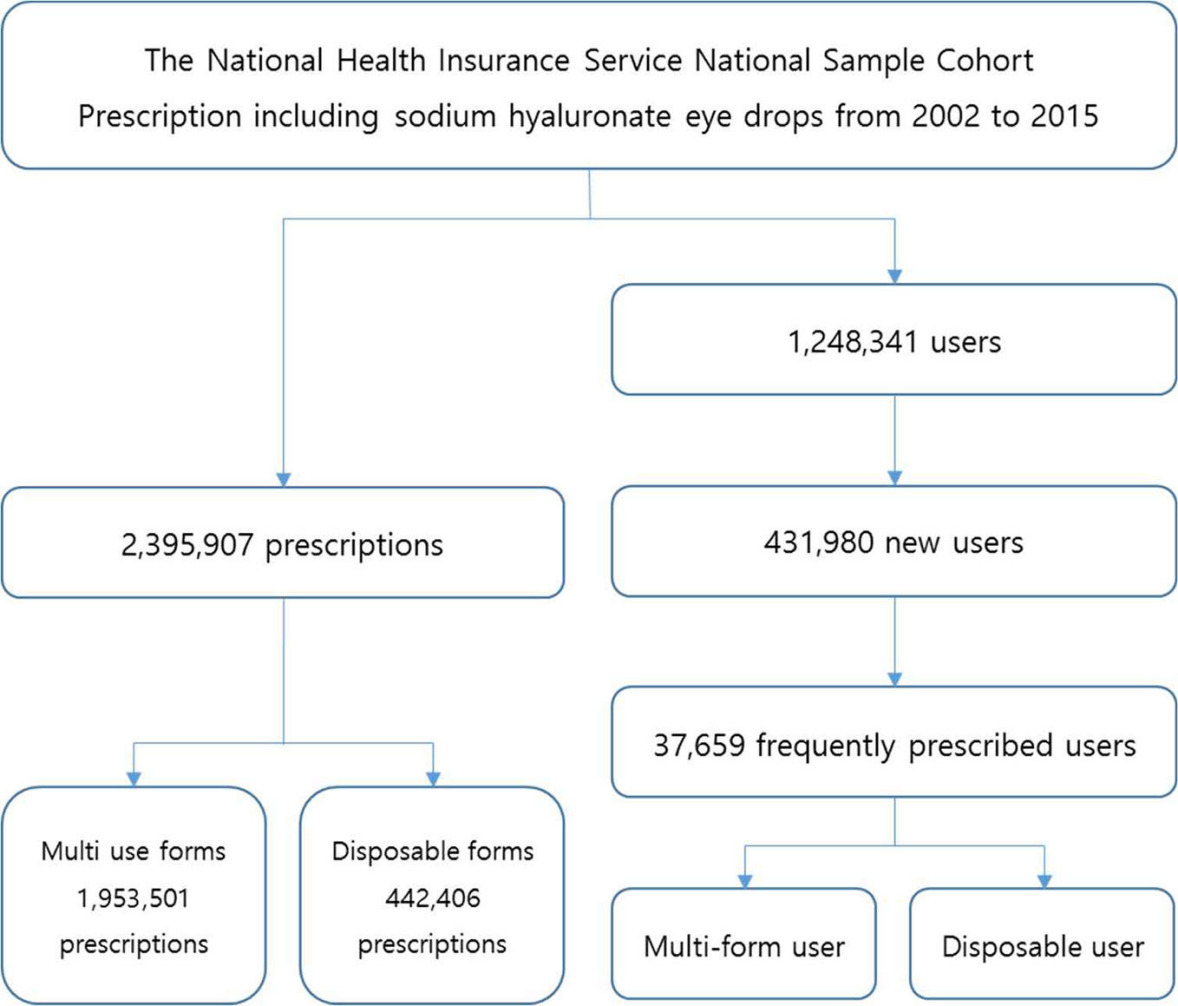
Flowchart of the study design.

**Figure 1** presents the flowchart for this study. Prescriptions including sodium hyaluronate eye drops and patients who have been prescribed the medicine were the subjects of this study. Specifically, we separated prescriptions including sodium hyaluronate eye drops, and then identified various types of eye drop users (general users, new users, and frequently prescribed users) to understand trends in the utilization of sodium hyaluronate eye drops in the South Korean market. General users were defined as patients who were prescribed eye drops in a certain year, while new users were defined as users who had not been prescribed any sodium hyaluronate eye drops in the 2 years prior to a certain year. Finally, frequently prescribed users were defined as patients who had been prescribed any sodium hyaluronate eye drops 10 times or more during the study period.

Currently, two types of eye drops are available on the market: disposable and multiuse forms. Disposable eye drops were defined as medicines with packing specifications less than 1 ml without the presence of ophthalmic preservatives, while multiuse forms were defined as medicines with packing specifications greater than 1 ml with the presence of ophthalmic preservatives. With these definitions, separated information on prescriptions, patients, and health-care institutions was obtained. Additionally, frequently prescribed users were categorized into two groups: the disposable user group and the multiuse form user group.

### Variables and Data Analysis

Descriptive statistics were used to examine the characteristics of prescriptions, patients (including general users, new users, and frequently prescribed users), and health-care institutions. At the patient level, information on the sociodemographic characteristics of the patients, including sex, age, and types of health insurance, were collected. Types of health insurance included the NHIS for the population and the Medical Aid Program for low-income household. Members of the NHIS were categorized into two types: self-employee and the employee. In South Korea, the premium that the member of NHIS pay are calculated based on their income and assets. Thus, the premium was used to proxy their income level, and separated into quintiles. The first quintile indicates the lowest income, while the fifth quintile indicates the highest income.

At the health-care institution level, information on the location and type of institution were obtained. First, the location was divided into several regions based on the administrative district. In the end, four regions were created, including Seoul, metropolitan excluding Seoul, city excluding metropolitan, and rural. Second, the health-care institutions were categorized as primary-, secondary-, tertiary-, and quaternary-care institutions (Medical Services Act, 2020): primary-care institutions include clinic-level institutions that provide health-care services to outpatients; secondary- and tertiary-care institutions include hospital-level institutions that provide services primarily to inpatients; and quaternary-care institutions include superior general hospitals, designated by the Minister of Health and Welfare, that provide service requiring expertise for treating serious disease. Finally, odds ratio with 95% confidence interval were calculated to examine the association between demographic variables (age, sex, region, types of insurance, and income level) and prescription categories. Data management and analysis were performed using R statistical software (V.3.4.4). Particularly, “epitools” package was used to obtain odds ratio. P values less than 0.05 were considered to be significant.

### Ethical Statement

A deidentified secondary dataset was used for this study, indicating that the study was exempted from review by the Institutional Review Board (IRB) of Ewha Woman's University (IRB No ewha-201903-0005-01).

### Patient and Public Involvement

No patients were involved in developing or conducting the research. Thus, the dissemination of the results of the research directly to the eligible participants is not required.

## Results

### Prescriptions of Sodium Hyaluronate Eye Drops

**Table 1** shows prescriptions including sodium hyaluronate eye drops from 2002 to 2015 in South Korea. The number of prescriptions has increased from 39,838 prescriptions in 2002 to 276,537 prescriptions in 2015 with a 15% compound annual growth rate (CAGR) for period 2002–2015. During the study period, prescriptions of sodium hyaluronate eye drops were mainly prescribed by ophthalmology (95%). In this study, eye drops were categorized into two types: multiuse and disposable forms. Disposable forms first became available on the market in 2003, and the number of their prescriptions increased steeply and reached 103,231 in 2015, with a 55% CAGR for period 2004–2015. The share of disposable forms in total prescriptions also increased from 1% in 2005 to 37% in 2015. The price of disposable forms is expensive than that of multiuse forms. Thus, pharmaceutical expenditure for disposable forms is quite increased and accounts for a large part of the expenditure for sodium hyaluronate eye drops. For instance, the total pharmaceutical expenditure for eye drops was 143 thousand United Stated Dollar (USD) in 2002. However, the expenditure increased to 2.593 million USD in 2015, with a 23% CAGR for period 2002–2015, and 65% of the expenditure was explained by disposable prescriptions. Given that the NHIS-NSC is composed of 2% of the total population, we estimated total market for sodium hyaluronate eye drops in South Korea. The market was estimated 7 million USD in 2002 and reached 130 million USD in 2015. To understand the impact of eye drops in health system, we provided total benefits provided by the NHI (Health Insurance Review and Assessment Service, 2020). The proportion of eye drops accounted for 0.06% and 0.34% in 2002 and 2015, respectively.

**Table 1:** Characteristics of prescriptions including sodium hyaluronate eye drop over time.

	2002	2003	2004	2005	2006	2007	2008	2009	2010	2011	2012	2013	2014	2015
**Total prescriptions**	39,838	54,539	77,403	98,961	124,346	148,861	168,245	193,632	219,421	230,691	242,299	254,125	267,009	276,537
Ophthalmology	37,960 (95%)	51,730 (95%)	73,444 (95%)	93,675 (95%)	117,015 (94%)	139,854 (94%)	158,286 (94%)	182,465 (94%)	205,838 (94%)	214,887 (93%)	226,382 (93%)	236,916 (93%)	249,083 (93%)	257,866 (93%)
Internal medicine	1,022 (3%)	1,218 (2%)	1,945 (3%)	2,626 (3%)	3,731 (3%)	4,624 (3%)	5,110 (3%)	5,827 (3%)	7,171 (3%)	8,217 (4%)	8,246 (3%)	9,134 (4%)	9,495 (4%)	10,168 (4%)
Others	856 (2%)	1,591 (3%)	2,014 (3%)	2,660 (3%)	3,600 (3%)	4,383 (3%)	4,849 (3%)	5,340 (3%)	6,412 (3%)	7,587 (3%)	7,671 (3%)	8,075 (3%)	8,431 (3%)	8,503 (3%)
**Multi-use forms**	39,838 (100%)	54,522 (100%)	77,074 (100%)	98,244 (99%)	121,153 (97%)	142,861 (96%)	154,504 (92%)	170,443 (88%)	188,040 (86%)	190,188 (82%)	184,734 (76%)	182,085 (72%)	176,509 (66%)	173,306 (63%)
Ophthalmology	37,960 (95%)	51,713 (95%)	73,126 (95%)	92,977 (95%)	113,907 (94%)	134,073 (94%)	144,961 (94%)	159,939 (94%)	175,572 (93%)	176,192 (93%)	171,266 (93%)	168,510 (93%)	163,183 (92%)	160,316 (93%)
Internal medicine	1,022 (3%)	1,218 (2%)	1,940 (3%)	2,616 (3%)	3,679 (3%)	4,543 (3%)	4,907 (3%)	5,466 (3%)	6,620 (4%)	7,420 (4%)	7,092 (4%)	7,159 (4%)	6,962 (4%)	7,138 (4%)
Others	856 (2%)	1,591 (3%)	2,008 (3%)	2,651 (3%)	3,567 (3%)	4,245 (3%)	4,636 (3%)	5,038 (3%)	5,848 (3%)	6,576 (3%)	6,376 (3%)	6,416 (4%)	6,364 (4%)	5,852 (3%)
**Disposable forms**	–	17 (0%)	329 (0%)	717 (1%)	3,193 (3%)	6,000 (4%)	13,741 (8%)	23,189 (12%)	31,381 (14%)	40,503 (18%)	57,565 (24%)	72,040 (28%)	90,500 (34%)	103,231 (37%)
Ophthalmology	–	17 (100%)	318 (97%)	698 (97%)	3,108 (97%)	5,781 (96%)	13,325 (97%)	22,526 (97%)	30,266 (96%)	38,695 (96%)	55,116 (96%)	68,406 (95%)	85,900 (95%)	97,550 (94%)
Internal medicine	–	–	5 (2%)	10 (1%)	52 (2%)	81 (1%)	203 (1%)	361 (2%)	551 (2%)	797 (2%)	1,154 (2%)	1,975 (3%)	2,533 (3%)	3,030 (3%)
Others	–	–	6 (2%)	9 (1%)	33 (1%)	138 (2%)	213 (2%)	302 (1%)	564 (2%)	1,011 (2%)	1,295 (2%)	1,659 (2%)	2,067 (2%)	2,651 (3%)
**Expenditure (1,000 USD)**	143	190	289	397	552	708	870	1,068	1,329	1,528	1,617	1,918	2,278	2,593
Multi-use forms	143 (100%)	190 (100%)	284 (98%)	382 (96%)	495 (90%)	600 (85%)	661 (76%)	733 (69%)	844 (64%)	872 (57%)	758 (47%)	795 (41%)	848 (37%)	897 (35%)
Disposable	–	–	5 (2%)	15 (4%)	57 (10%)	108 (10%)	209 (24%)	335 (31%)	485 (36%)	656 (43%)	859 (53%)	1,123 (59%)	1,430 (63%)	1,696 (65%)
**Estimated market (million USD)**	7	10	14	20	28	35	44	53	66	76	81	96	114	130
**Total benefits provided by the NHI (million USD)**	11,391	12,523	13,691	15,304	17,865	20,479	21,983	24,973	28,163	30,046	31,111	33,218	35,487	38,108
**Percent of estimated market**	0.06%	0.08%	0.11%	0.13%	0.15%	0.17%	0.20%	0.21%	0.24%	0.25%	0.26%	0.29%	0.32%	0.34%

Total prescriptions are composed of multi-use and disposable forms.

Given the sample size (2% of the total population) of the NHIS-NSC, estimated market size was calculated.

Percent of estimated market was calculated using estimated market and total benefits provided by the NHI (HIRA, 2020).

United States Dollar to South Korean Won exchange rate was assumed to be 1,200.

Results were rounded.

### Characteristics of Sodium Hyaluronate Eye Drop Users

#### General Users

**Table 2** presents the characteristics of sodium hyaluronate eye drop users over time. The prevalence of eye drop users increased from 2,562/100,000 persons in 2002 to 14,372/100,000 persons in 2015, with a 13% CAGR for period 2002–2015. More female users than male users were reported during the study period, while the proportion of female users slightly declined from 69% in 2002 to 66% in 2015. The average age of users has continuously increased. For instance, the average age of users in 2002 was 42.1 years old, while that of users in 2015 was 51.9 years old. Users were sorted by their residence, including Seoul, metropolitan excluding Seoul, city, and rural, and the residence proportions remained steady. The proportion in the first quintile (the lowest income) increased from 12% in 2002 to 16% in 2015, while the proportion in the fifth quintile (the highest income) remained steady during the study period.

**Table 2:** Characteristics of general users prescribed sodium hyaluronate eye drops over time.

	2002	2003	2004	2005	2006	2007	2008	2009	2010	2011	2012	2013	2014	2015
General users	24,805	33,626	46,440	58,287	68,991	80,978	88,583	99,709	111,844	116,520	122,295	126,647	132,566	137,050
Prevalence (100,000 person)	2,562	3,435	4,695	5,829	6,899	8,098	8,914	10,092	11,386	11,935	12,602	13,130	13,823	14,372
**Sex**														
Female (%)	17,066 (69%)	22,765 (68%)	31,301 (67%)	38,644 (66%)	45,534 (66%)	53,445 (66%)	58,288 (66%)	65,210 (65%)	73,146 (65%)	76,903 (66%)	80,226 (66%)	83,334 (66%)	87,228 (66%)	90,042 (66%)
**Age**														
Mean (SD)	42.1 (17.7)	43.6 (18.0)	44.7 (18.1)	45.7 (18.5)	46.9 (19.0)	47.5 (19.3)	48.8 (19.3)	49.3 (19.4)	49.3 (19.7)	50.0 (19.6)	50.2 (19.7)	50.8 (19.7)	51.5 (19.6)	51.9 (19.5)
**Region**														
Seoul	5,561 (22%)	7,511 (22%)	10,321 (22%)	12,578 (22%)	14,533 (21%)	16,616 (21%)	18,038 (20%)	20,138 (20%)	22,671 (20%)	23,197 (20%)	23,843 (19%)	24,641 (19%)	26,006 (20%)	26,883 (20%)
Metropolitan excluding Seoul	6,798 (27%)	8,879 (26%)	12,234 (26%)	15,637 (27%)	18,188 (26%)	21,310 (26%)	23,536 (27%)	26,414 (26%)	29,867 (27%)	30,989 (27%)	32,553 (27%)	33,988 (27%)	36,092 (27%)	37,480 (27%)
City	5,382 (22%)	7,425 (22%)	10.296 (22%)	12,788 (22%)	14,822 (21%)	17,710 (22%)	18,834 (21%)	21,177 (21%)	23,801 (21%)	25,119 (22%)	26.662 (22%)	27,978 (22%)	29,280 (22%)	29,902 (22%)
Rural	7,064 (28%)	9,811 (29%)	13,589 (29%)	17,284 (30%)	21,448 (31%)	25,342 (31%)	28,175 (32%)	31,980 (32%)	35,505 (32%)	37,215 (32%)	39,237 (32%)	40,040 (32%)	41,188 (31%)	42,785 (31%)
**Types**														
Self-employed	10,940 (44%)	14,746 (44%)	19,463 (42%)	23,176 (40%)	24,883 (36%)	28,005 (35%)	29,326 (33%)	32,066 (32%)	35,388 (32%)	35,267 (30%)	35,567 (29%)	35,335 (28%)	35,780 (27%)	36,224 (26%)
The employee	13,823 (56%)	18,844 (56%)	26,885 (58%)	34,431 (59%)	40,354 (58%)	48,452 (60%)	54,183 (61%)	61,945 (62%)	70,712 (63%)	75,273 (65%)	80,720 (66%)	85,361 (67%)	90,819 (69%)	94,609 (69%)
Medical aid	42 (0%)	36 (0%)	92 (0%)	680 (1%)	3,754 (5%)	4,521 (6%)	5,074 (6%)	5,698 (6%)	5,744 (5%)	5,980 (5%)	6,008 (5%)	5,951 (5%)	5,967 (5%)	6,217 (5%)
**Income level**														
The first	3,015 (12%)	4,678 (14%)	6,224 (14%)	8,766 (15%)	9,697 (15%)	10,890 (14%)	11,324 (14%)	13,153 (14%)	14,702 (14%)	15,966 (15%)	16,723 (14%)	18,373 (15%)	19,000 (15%)	20,671 (16%)
The second	3,471 (14%)	4,468 (13%)	6,831 (15%)	7,029 (12%)	9,532 (15%)	10,285 (14%)	11,944 (14%)	13,404 (14%)	15,608 (15%)	15,510 (14%)	17,382 (15%)	16.910 (14%)	18,228 (15%)	18,228 (14%)
The third	5,140 (21%)	5,945 (18%)	8,044 (18%)	10,559 (19%)	10,383 (16%)	13,397 (18%)	14,198 (17%)	16,374 (18%)	17,945 (17%)	18,992 (17%)	19,278 (17%)	20,133 (17%)	21,325 (17%)	21,757 (17%)
The fourth	5,559 (23%)	8,195 (25%)	10,736 (23%)	12,504 (22%)	14,968 (23%)	17,403 (23%)	18,950 (23%)	20,970 (23%)	24,012 (23%)	24,734 (23%)	25,823 (22%)	26,825 (22%)	27,739 (22%)	28,792 (22%)
The fifth	7,306 (30%)	9,970 (30%)	14,036 (31%)	18,149 (32%)	19,972 (31%)	23,748 (31%)	26,329 (32%)	29,272 (31%)	32,952 (31%)	34,411 (31%)	36,138 (31%)	37,498 (31%)	39,349 (31%)	40,377 (31%)

Results were rounded.

#### New Users

**Table 3** shows the characteristics of new sodium hyaluronate eye drop users over time. In this study, new eye drop users were defined as those who had not been prescribed any sodium hyaluronate eye drops in the 2 years prior to the first prescription observed in the cohort dataset for the period from 1 January 2002 to 31 December 2015. Thus, new eye drop users in 2002 and 2003 could not be defined in this study. The incidence of eye drop users remained steady from 3,348/100,000 persons in 2002 to 3,094/100,000 persons in 2015. Similar to general users presented in **Table 1**, more female new users than male users were reported during the study period, while the proportion of female users declined from 65% in 2002 to 54% in 2015. The average age of new users and their residence have remained steady. However, the proportion in Seoul slightly declined, while that in metropolitan areas excluding Seoul slightly increased. The proportion in the first quintile (the lowest income) increased from 14% in 2002 to 16% in 2015, while that in the fifth quintile (the highest income) decreased from 30% in 2004 to 28% in 2015.

**Table 3:** Characteristics of new users prescribed sodium hyaluronate eye drops over time.

	2002	2003	2004	2005	2006	2007	2008	2009	2010	2011	2012	2013	2014	2015
New users			33,121	36,772	38,634	39,874	38,670	39,346	40,124	37,063	35,223	32,428	31,224	29,501
Incidence (100,000 person)			3,348	3,677	3,863	3,987	3,891	3,982	4,085	3,796	3,629	3,362	3,256	3,094
**Sex**														
Female (%)			21,601 (65%)	23,146 (62%)	24,024 (62%)	24,381 (61%)	23,309 (60%)	23,252 (59%)	23,401 (58%)	21,626 (58%)	20,016 (56%)	18,294 (56%)	17,224 (55%)	16,125 (54%)
**Age**														
Mean (SD)			42.1 (17.7)	42.5 (17.9)	42.9 (17.9)	43.1 (18.5)	43.7 (19.2)	43.0 (19.3)	43.5 (19.3)	43.1 (19.5)	42.1 (19.6)	42.2 (19.5)	41.6 (19.5)	41.7 (19.7)
**Region**														
Seoul			7,256 (22%)	7,796 (21%)	7,940 (21%)	8,026 (20%)	7,770 (20%)	7,728 (20%)	8,010 (20%)	7,264 (20%)	6,702 (19%)	6,087 (19%)	6,113 (20%)	5,715 (19%)
Metropolitan excluding Seoul			8,618 (26%)	9,708 (26%)	9,891 (26%)	10,405 (26%)	10,305 (27%)	10,485 (27%)	10,899 (27%)	10,046 (27%)	9,602 (27%)	9,028 (28%)	8,859 (28%)	8,512 (29%)
City			7,389 (22%)	8,063 (22%)	8,391 (22%)	8,790 (22%)	8,089 (21%)	8,394 (21%)	8,564 (21%)	8,010 (22%)	7,705 (22%)	7,019 (22%)	6,832 (22%)	6,137 (21%)
Rural			9,858 (30%)	11,205 (30%)	12,412 (32%)	12,653 (32%)	12,506 (32%)	12,739 (32%)	12,651 (32%)	11,743 (32%)	11,214 (32%)	10,294 (32%)	9,420 (30%)	9,137 (31%)
**Types**														
Self-employed			14,035 (42%)	14,888 (40%)	13,854 (36%)	14,217 (36%)	13,284 (34%)	13,284 (34%)	13,177 (33%)	11,830 (32%)	10,492 (30%)	9,337 (29%)	8,664 (28%)	7,999 (27%)
The employee			18,998 (57%)	2,259 (58%)	21,822 (56%)	23,390 (59%)	23,470 (61%)	24,169 (61%)	25,338 (63%)	23,840 (64%)	23,471 (67%)	21,993 (68%)	21,637 (69%)	20,599 (70%)
Medical aid			88 (0%)	625 (2%)	2,958 (8%)	2,267 (6%)	1,916 (5%)	1,893 (5%)	1,609 (4%)	1,393 (4%)	1,260 (4%)	1,058 (3%)	923(3%)	903 (3%)
**Income level**														
The first			4,423 (14%)	5,518 (15%)	5,420 (15%)	5,464 (15%)	5,063 (14%)	5,302 (14%)	5,331 (14%)	5,189 (15%)	4,888 (15%)	4,872 (16%)	4,520 (15%)	4,481 (16%)
The second			4,965 (15%)	4,558 (13%)	5,435 (15%)	5,283 (14%)	5,656 (16%)	5,694 (15%)	6,029 (16%)	5,407 (15%)	5,332 (16%)	4,676 (15%)	4,682 (16%)	4,165 (15%)
The third			5,858 (18%)	6,788 (19%)	5,873 (17%)	6,985 (19%)	6,532 (18%)	7,003 (19%)	7,010 (18%)	6,545 (19%)	6,030 (18%)	5,669 (18%)	5,457 (18%)	5,215 (18%)
The fourth			7,679 (23%)	7,943 (22%)	8,200 (23%)	8,641 (23%)	8,423 (23%)	8,444 (23%)	8,792 (23%)	8,060 (23%)	7,684 (23%)	6,990 (22%)	6,831 (23%)	6,474 (23%)
The fifth			9,765 (30%)	10,945 (31%)	10,353 (29%)	10,859 (29%)	10,712 (29%)	10,655 (29%)	11,002 (29%)	10,131 (29%)	9,702 (29%)	8,895 (29%)	8,542 (28%)	8,005 (28%)

Results were rounded.

#### Frequently Prescribed Users

**Figure 2:**
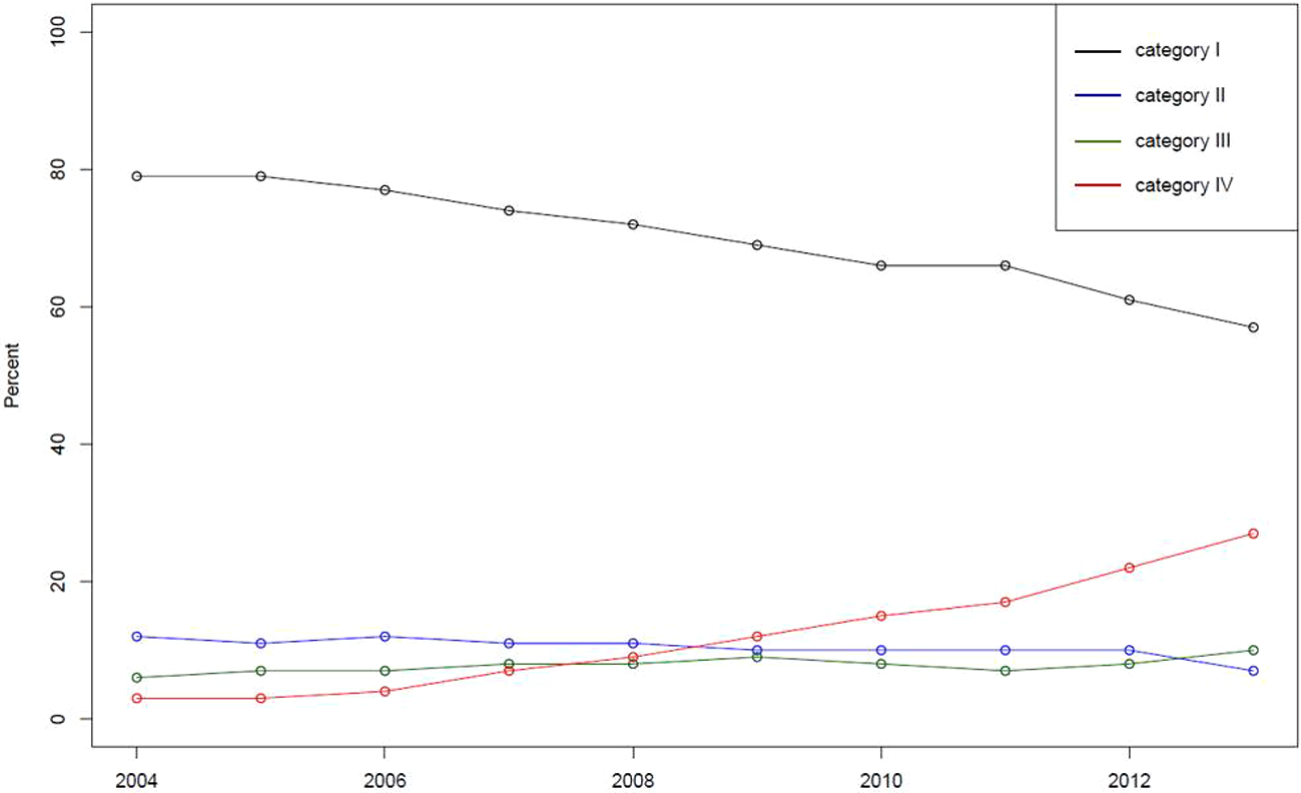
Proportion of frequently prescribed users over time sorted by four categories based on the proportion of disposable forms in prescriptions. Note: Category I indicates the group in which the proportion using disposable forms is less than one-quarter. Category II indicates the proportion between one-quarter and one-half. Category III indicates the proportion between one-half and three-fourths. Category IV indicates the proportion more than three-quarters.

**Figure 2** presents proportion of frequently prescribed users over time sorted by four categories based on the proportion of disposable forms in prescriptions. Frequently prescribed users were defined as those who had been defined as new users in this study and prescribed any sodium hyaluronate eye drops 10 times or more during the study period. Category I means the group in which the proportion using disposable forms is less than one-quarter, indicating multiuse form users, while category II means the group in which the proportion using disposable forms is more than one-quarter but less than one-half. Similarly, category IV means the group in which the proportion using disposable forms is more than three-fourths, indicating disposable form users, while category III means the group in which the proportion using disposable forms is more than one-half but less than three-fourths.

The proportion of category IV users (or disposable users) has increased, while that of category I users (or multiuse form users) has decreased during the study period. The proportion of disposable user and multiuse form users in 2013 was 27% and 57%, respectively, indicating that the number of multiuse form users was still higher than that of disposable users among frequently prescribed users. Then, frequently prescribed users in 2005 and 2010 were selected to examine the odds ratio between demographic variables (age, sex, region, types of insurance, and income level) and prescription categories. **Table 4** presents odds ration with 95% confidence interval for 2005 and 2010, using a reference category for each subgroup. In the table, sex and age of users presented the association with prescription categories.

**Table 4:** Odds ratio with confidence interval between demographic variables and prescription categories in 2005 and 2010.

	Odds ratio (95% CI)	
	2005	2010
**Sex (ref male)**		
Female	2.13 (1.50–3.05)	2.02 (1.55–2.62)
**Age (ref 60 <)**		
<30	26.06 (17.05–39.82)	38.38 (25.81–57.09)
30–60	4.62 (3.11–8.59)	7.42 (5.42–5.78)
**Region (ref rural)**		
Seoul	1.62 (1.12–2.37)	1.62 (1.18–2.22)
Metropolitan excluding Seoul	1.01 (0.68–1.51)	1.17 (0.85–1.62)
City	1.10 (0.73–1.64)	1.39 (1.02–1.89)
**Types (ref medical aid)**		
Self-employed	2.15 (0.99–4.69)	1.08 (0.70–1.66)
The employee	2.00 (0.92–4.33)	1.44 (0.97–2.14)
**Income level (ref the first)**		
The second	0.89 (0.52–1.54)	1.63 (1.04–2.55)
The third	0.91 (0.56–1.47)	1.34 (0.86–2.09)
The fourth	0.94 (0.59–1.47)	1.47 (0.97–2.23)
The fifth	1.14 (0.76–1.71)	1.25 (0.83–1.87)

### Characteristics of Institutions Prescribing Sodium Hyaluronate Eye Drops

**Table 5** presents the characteristics of prescriptions sorted by health-care institutions. Overall, the majority of prescriptions were prescribed in primary-care institutions. For instance, 92, 1, 4, and 4% of prescriptions were from primary-, secondary-, tertiary-, and quaternary-care institutions, respectively, in 2002, and the trends remained steady (90, 3, 4, and 3%, respectively) for the aforementioned institutions. The prescriptions were divided into multiuse and disposable forms, and then the proportions of types of institutions and their location in each category were presented in the same table. For instance, the proportions of primary-, secondary-, tertiary-, and quaternary-care institutions prescribing disposable forms were 83, 7, 5, and 5%, respectively, in 2015. Similarly, the proportions of the aforementioned institutions prescribing multiuse forms were 93, 3, 2, and 1%, respectively, in the same year.

**Table 5:** Characteristics of prescriptions sorted by health-care institutions over time.

	2002	2003	2004	2005	2006	2007	2008	2009	2010	2011	2012	2013	2014	2015
**Total prescription**	**39,838**	**54,539**	**77,403**	**98,961**	**124,346**	**148,861**	**168,245**	**193,632**	**219,421**	**230,691**	**242,299**	**254,125**	**267,009**	**276,537**
Types														
Quaternary	1,437 (4%)	1,930 (4%)	2,461 (3%)	3,010 (3%)	3,684 (3%)	4,065 (3%)	4,695 (3%)	5,760 (3%)	6,389 (3%)	6,411 (3%)	6,494 (3%)	6,642 (3%)	7,461 (3%)	7,794 (3%)
Tertiary	1,414 (4%)	2,169 (4%)	3,086 (4%)	4,016 (4%)	4,850 (4%)	5,378 (4%)	6,342 (4%)	6,980 (4%)	7,629 (4%)	7,306 (4%)	7,742 (4%)	8,240 (4%)	9,036 (4%)	9,259 (4%)
Secondary	317 (1%)	1,529 (3%)	2,465 (3%)	3,471 (4%)	4,689 (4%)	5,015 (3%)	5,612 (3%)	5,789 (3%)	7,423 (3%)	8,245 (3%)	9,277 (3%)	10,961 (3%)	10,969 (3%)	11,471 (3%)
Primary	36,670 (92%)	48,911 (90%)	69,391(90%)	88,464 (89%)	111,123 (89%)	134,403 (90%)	151,596 (90%)	175,103 (90%)	197,980 (90%)	208,729 (90%)	218,786 (90%)	228,282 (90%)	239,543 (90%)	248,013 (90%)
**Multi-use**	**39,838 (100%)**	**54,522 (100%)**	**77,074 (100%)**	**98,244 (99%)**	**121,153 (97%)**	**142,861 (96%)**	**154,504 (92%)**	**170,443 (88%)**	**188,040 (86%)**	**190,188 (82%)**	**184,734 (76%)**	**182,085 (72%)**	**176,509 (66%)**	**173,306 (63%)**
Types														
Quaternary	1,437 (4%)	1,930 (4%)	2,412 (3%)	2,872 (3%)	3,258 (3%)	3,339 (2%)	3,481 (2%)	3,685 (2%)	3,891 (2%)	3,735 (2%)	3,411 (2%)	2,883 (2%)	2,620 (1%)	2,415 (1%)
Tertiary	1,414 (4%)	2,169 (4%)	3,053 (4%)	3,937 (4%)	4,296 (4%)	4,414 (3%)	4,919 (3%)	4,947 (3%)	5,250 (3%)	4,814 (3%)	4,550 (2%)	4,377 (2%)	4,048 (2%)	3,804 (2%)
Secondary	317 (1%)	1,529 (3%)	2,447 (3%)	3,427 (3%)	4,426 (4%)	4,562 (3%)	4,596 (3%)	4,395 (3%)	5,418 (3%)	5,743 (3%)	5,584 (3%)	6,043 (3%)	5,484 (3%)	5,199 (3%)
Primary	36,670 (92%)	48,894 (90%)	69,162 (90%)	88,008 (90%)	109,173 (90%)	130,546 (91%)	141,508 (92%)	157,416 (92%)	173,481 (92%)	175,896 (92%)	171,189 (93%)	168,782 (93%)	164,357 (93%)	161,888 (93%)
Region														
Seoul	10,229 (26%)	13,679 (25%)	18,754 (24%)	23,205 (24%)	27,531 (23%)	31,070 (22%)	32,804 (21%)	35,496 (21%)	38,196 (20%)	38,391(20%)	36,253 (20%)	34,851 (19%)	33,195 (19%)	32,602 (19%)
Metropolitan excluding Seoul	9,898 (25%)	13,327 (24%)	19,011 (25%)	24,747 (25%)	29,233 (24%)	34,554 (24%)	36,765 (24%)	40,679 (24%)	45,710 (24%)	45,637 (24%)	44,342 (24%)	43,046 (24%)	41,899 (24%)	41,276 (24%)
City	10,053 (25%)	13,749 (25%)	19,245 (25%)	24,138 (25%)	29,243 (24%)	34,352 (24%)	36,456 (24%)	40,422 (24%)	44,541 (24%)	44,950 (24%)	44,180 (24%)	44,951 (25%)	43,476 (25%)	42,012 (24%)
Rural	9,658 (24%)	13,767 (25%)	20,064 (26%)	26,154 (27%)	35,146 (29%)	42,885 (30%)	48,479 (31%)	53,846 (32%)	59,593 (32%)	61,210 (32%)	59,959 (32%)	59,237 (33%)	57,939 (33%)	57,416 (33%)
**Disposal**	**-**	**17 (0%)**	**329 (0%)**	**717 (1%)**	**3,193 (3%)**	**6,000 (4%)**	**13,741 (8%)**	**23,189 (12%)**	**31,381 (14%)**	**40,503 (18%)**	**57,565 (24%)**	**72,040 (28%)**	**90,500 (34%)**	**103,231 (37%)**
Types														
Quaternary	–	–	49 (15%)	138 (19%)	426 (13%)	726 (12%)	1,214 (9%)	2,075 (9%)	2,498 (8%)	2,676 (7%)	3,083 (5%)	3,759 (5%)	4,841 (5%)	5,379 (5%)
Tertiary	–	–	33 (10%)	79 (11%)	554 (17%)	964 (16%)	1,423 (10%)	2,033 (9%)	2,379 (8%)	2,492 (6%)	3,192 (6%)	3,863 (5%)	4,988 (6%)	5,455 (5%)
Secondary	–	–	18 (5%)	44 (6%)	263 (8%)	453 (8%)	1,016 (7%)	1,394 (6%)	2,005 (6%)	2,502 (6%)	3,693 (6%)	4,918 (7%)	5,485 (6%)	6,272 (7%)
Primary	–	17 (100%)	229 (70%)	456 (64%)	1,950 (61%)	3,857 (64%)	10,088 (73%)	17,687 (76%)	24,499 (78%)	32,833 (81%)	47,597 (83%)	59,500 (83%)	75,186 (83%)	86,125 (83%)
Region														
Seoul	–	1 (6%)	125 (38%)	226 (32%)	1,030 (32%)	1,954 (33%)	3,792 (28%)	6,461 (28%)	8,889 (28%)	10,985 (27%)	15,178 (26%)	19,731 (27%)	23,937 (26%)	26,081 (25%)
Metropolitan excluding Seoul	–	9 (53%)	50 (15%)	170 (24%)	749 (23%)	1,438 (24%)	3,223 (23%)	5,018 (22%)	7,047 (22%)	9,059 (22%)	13,160 (23%)	17,017 (24%)	22,600 (25%)	25,712 (25%)
City	–	4 (23%)	67 (20%)	162 (23%)	805 (25%)	1,441 (24%)	3,813 (28%)	6,562 (28%)	8,752 (28%)	11,552 (29%)	16,172 (28%)	18,842 (26%)	23,396 (26%)	26,993 (26%)
Rural	–	3 (18%)	87 (26%)	159 (22%)	609 (19%)	1,167 (19%)	2,913 (21%)	5,148 (22%)	6,693 (21%)	8,907 (22%)	13,055 (23%)	16,450 (23%)	20,567 (23%)	24,445 (24%)

**Figure 3:**
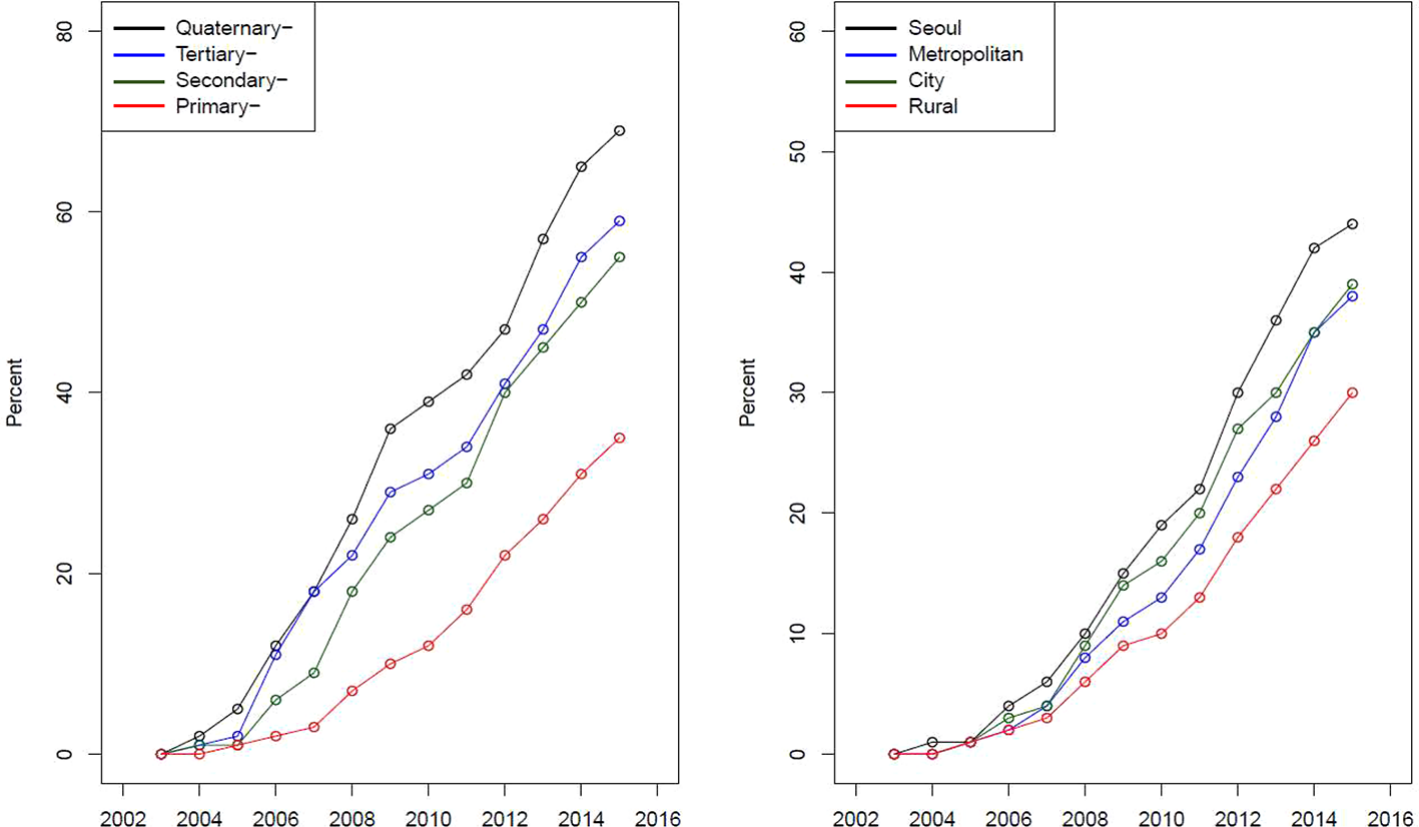
Proportion of disposable forms over time sorted by types of institutions and regions.

**Figure 3** presents a curve for the proportion of disposable forms sorted by types of institutions and regions. The proportion of the prescription of disposable forms has increased for the four types of institutions. However, the speed of the increase in the proportion of the prescription of disposable forms was different among types of institutions. Similarly, the proportion of the prescription of disposable forms has increased for the four regions, with variation in the speed of increase.

## Discussion

In this study, we evaluated the trends in the utilization of sodium hyaluronate eye drops in South Korea from a large-scale population-based dataset and analyzed prescriptions, patients, and health-care institutions, which were separated by disposable and multiuse forms, to discuss the impact of the introduction of expensive disposable forms from the perspective of health financing and equity in utilization.

### The Prevalence of Prescriptions of Sodium Hyaluronate

The prevalence of prescriptions of sodium hyaluronate eye drops increased from 2,562/100,000 persons in 2002 to 14,732/100,000 persons in 2015. It is interesting to compare the prevalence of prescriptions of sodium hyaluronate reported in this study directly with those reported in other studies on the prevalence of DED (Han et al., 2011; Ahn et al., 2014). For instance, Ahn *et al*. (2014) reported that the overall prevalence of DED diagnosis and the prevalence of dry eye symptoms in South Korea were 8 and 14.4%, respectively, in 2010–11 (Ahn et al., 2014). The prevalence of prescriptions of sodium hyaluronate eye drops reported in this study was 11.93% in 2010, which lies in the middle between the overall prevalence and the symptom-based prevalence.

The variance in prevalence could be explained by two factors: the definition of DED and the study population. Given that the majority of patients were prescribed eye drops for dry eye syndrome, we defined DED patients as those who were prescribed sodium hyaluronate eye drops. However, Ahn et al. (2014) defined DED patients as those who were clinically diagnosed and those who had at least one symptom of dry eye, which was based on a self-reported survey, for overall prevalence and symptom-based prevalence, respectively (Ahn et al., 2014). Note the discrepancy between objectively measured diagnosis and subjectively self-reported symptoms in DED (Hua et al., 2014). Additionally, Ahn et al. (2014) randomly selected 11,666 subjects, ranging in age from 19 to 95, from the Korea National Health and Nutrition Examination Survey (Ahn et al., 2014). Thus, adolescents under 18 years old were excluded from the study (Kweon et al., 2014).

We separated eye drop users into general users and new users to calculate the prevalence and incidence of prescriptions involving sodium hyaluronate eye drops and found interesting trends in these groups. First, the prevalence of prescriptions has continuously increased during the study period, while the incidence of prescriptions has remained steady. Second, there have been more female patients in terms of both prevalence and incidence. However, the proportion of male patients was higher in incidence than in prevalence. Third, the average age of users increased during the study period, while that of new users decreased. These differences might imply that the increased use of computers and smartphones and dry indoor air caused by air conditioners or fan heaters is closely related to the increased incidence of prescriptions, particularly for males and young generations (McMonnies et al., 1998; Blehm et al., 2005; Moon et al., 2014; Park et al., 2014; Moon et al., 2016).

### Impact of the Introduction of Disposable Forms on Health Financing and Equity in Utilization

Ophthalmic preservatives have been understood as a risk factor for corneal and conjunctival inflammation (Messmer, 2015; Foulks et al., 2015). Thus, a disposable form had been available on the South Korean market to address the issue, particularly for patients with glaucoma, patients who need multiple eye drops, and patients wearing contact lenses.

We found that prescriptions for high priced disposable forms have steadily increased, while prescriptions for multiuse forms have decreased since 2012. Currently, pharmaceutical expenditure for disposable forms accounts for two-thirds of the total expenditure. For instance, the total pharmaceutical expenditure for sodium hyaluronate eye drops was 2.6 million USD, which is composed of 0.9 million (35%) USD and 1.7 million (65%) USD for multiuse and disposable forms, respectively. Furthermore, the proportion of disposable users among frequently prescribed users is increasing, indicating that the utilization of disposable forms might be an additional burden for the insurer from the perspective of health financing. For instance, the proportion of expenditure for sodium hyaluronate eye drops in total benefits, including medical service and prescription drugs, increased from 0.06% in 2002 to 0.34% in 2015. Thus, understanding for whom, under what conditions, and how eye drops in disposable forms are prescribed is essential to address their impacts on health financing.

Furthermore, introducing disposable forms of eye drops caused equity issue in utilization. In this study, we demonstrated that sex and age of users were associated with utilizing eye drops in disposable forms. Particularly, female users (reference male) and users under 30 years old (reference more than 60 years old) are more likely to be prescribed eye drops in disposable forms. DED is closely related to wearing contact lenses and eye makeup (Vehof et al., 2014; Messmer, 2015; Jones et al., 2017), which are more frequently observed among young women. Therefore, eye drops are likely to be prescribed to women and patients under 30 years old. Furthermore, eye drops in disposable forms are more convenient to use and carry, implying that women or patients under 30 years of age might prefer disposable forms to multiuse forms. Also, note that patients have to pay approximately 30% of the pharmaceutical expenditure under the NHIS in South Korea. Thus, patients who cannot afford eye drops, including geriatric patients and patients in low-income households, are less likely to be prescribed expensive eye drops in disposable form, indicating that being prescribed disposable forms might also be a burden for patients.

Sodium hyaluronate eye drops are prescription drugs that should be prescribed at health-care institutions. In this study, we found that the majority of eye drops (approximately 90%) were prescribed by primary-care institutions, indicating that DED was the most common disease causing patients to visit ophthalmology clinics. Furthermore, we found that the proportion of the prescription of disposable forms and their speed of adoption were different among various types of institutions. For instance, the proportion of the prescription of disposable forms was in the order of quaternary- tertiary-, secondary-, and primary-care institutions, which might imply that physicians at quaternary-care institutions were more likely to prescribe eye drops in the disposable form to their patients, including patients with glaucoma and patients who need multiple eye drops. However, note that the number of total prescriptions involving disposable and multiuse forms from quaternary-care institutions is much less than that of prescriptions from primary-care institutions, where the majority of eye drops are prescribed. Thus, the total impact of the introduction of disposable forms on health financing is greater in primary-care institutions.

We also found an interesting result in variance among four regions, including Seoul, metropolitan excluding Seoul, city, and rural area, for the prescription of disposable forms. The proportion of the four regions in prescriptions involving the disposable form was in the order of Seoul (38%), rural (26%), city (20%), and metropolitan excluding Seoul (15%) in 2004. However, the proportion of the Seoul area slightly decreased, while that of the rural area slightly increased during the study period. Finally, the proportion of the four regions in prescriptions involving the disposable form were converged to 25% in 2015. Similarly, we presented the proportion of disposable forms in the total prescriptions (**Figure 3**), and we found that the proportion was in the order of Seoul, city, metropolitan excluding Seoul, and rural. The figure implies that health-care institutions in Seoul or the city area are more likely to prescribe eye drops in disposable forms for their patients than those in rural areas.

### Limitations

This study has several limitations. First, this study used claims data that do not contain clinical information on or symptoms of patients who were prescribed sodium hyaluronate eye drops. This means that we could not assess prescriptions by disease severity. For instance, eye drops without ophthalmic preservatives are recommended for glaucoma patients, who need multiple eye drops (Foulks et al., 2015). Second, this study used prescriptions with sodium hyaluronate eye drops to approximate the prevalence of DED. Sodium hyaluronate eye drops could be prescribed for patients with Sjögren's syndrome or cutaneous mucosal syndrome (Steven-Jones syndrome), indicating that the prevalence of prescriptions involving eye drops and the prevalence of DED might differ. However, note that the majority of patients were prescribed eye drops for dry eye syndrome in South Korea (Kim et al., 2007; Kim, 2018). Finally, this study used claims data that do not include over-the-counter drugs for self-treatment. Similar to other countries (Clegg et al., 2006; Pucker et al., 2016), various forms of eye drops for DED are available without prescriptions in South Korea. Thus, this study would underestimate the utilization of eye drops or prevalence of DED.

## Conclusions

The prevalence of prescriptions of sodium hyaluronate eye drops has increased from 2,562/100,000 persons in 2002 to 14,732/100,000 persons in 2015, indicating that DED is the most common ocular disease causing patients to visit ophthalmology clinics. Furthermore, the increased use of computers and smartphones and dry indoor air caused by air conditioners or fan heaters is closely related to the incidence of prescriptions, particularly for males and young generations. For instance, the average age of new users slightly decreased from 42.1 years old in 2004 to 41.7 years old in 2015, and the average age of general users increased from 42.1 years old in 2002 to 51.9 years old in 2015. High-cost ophthalmic preservative-free eye drops were granted marketing authorization in 2003. Eye drops in disposable forms are safe more convenient to use and carry, implying that women or patients under 30 years of age might prefer disposable forms to multiuse forms. Thus, eye drops in disposable forms have expanded the eye drop market in South Korea and caused equity issues in utilization. The results indicate that the utilization of eye drops should be closely monitored from the perspectives of health equity. Particularly, geriatric patients and patients in low-income household should be accessible to eye drops in disposable forms when they need preservative-free eye drops.

## Data Availability Statement

The data that support the findings of this study are available from the NHIS but restrictions apply to the availability of these data, which were used under license for the current study, and so are not publicly available.

## Ethics Statement

The studies involving human participants were reviewed and approved by Ewha Woman's University. Written informed consent for participation was not required for this study in accordance with the national legislation and the institutional requirements. Written informed consent was not obtained from the individual(s) for the publication of any potentially identifiable images or data included in this article.

## Author Contributions

K-BS developed the concept of the paper, undertook the analysis, and wrote the manuscript.

## Funding

The author disclosed receipt of the following financial support for the research, authorship, and/or publication of this article: This work was supported by Taejoon Pharmaceutical Co., LTD. However, the funding source was not involved in the study design, collection, analysis and interpretation of data, writing of the report, and the decision to submit the article for publication.

## Conflict of Interest

The author declares that the research was conducted in the absence of any commercial or financial relationships that could be construed as a potential conflict of interest.
